# Fluctuation in the Assimilation of Problematic Experiences: A Case Study of Dynamic Systems Analysis

**DOI:** 10.3389/fpsyg.2018.01119

**Published:** 2018-08-15

**Authors:** Isabel Basto, William B. Stiles, Tiago Bento, Patrícia Pinheiro, Inês Mendes, Daniel Rijo, João Salgado

**Affiliations:** ^1^Center for Research in Neuropsychology and Cognitive Behavioral Intervention (CINEICC), Faculty of Psychology and Education Sciences, University of Coimbra, Coimbra, Portugal; ^2^University Institute of Maia (ISMAI), Maia, Portugal; ^3^Center of Psychology at University of Porto (CPUP), University of Porto, Porto, Portugal; ^4^Department of Psychology, Miami University, Oxford, OH, United States; ^5^Department of Psychology, Appalachian State University, Boone, NC, United States; ^6^Psychology Research Center (CIPsi), School of Psychology, University of Minho, Braga, Portugal; ^7^Royal Holloway, University of London, Egham, United Kingdom

**Keywords:** dynamic systems theory, assimilation model, instability, psychotherapeutic change process, depression, fluctuation, dynamic factor model

## Abstract

Dynamic systems theory suggests that instability can be a key element in the promotion of human change processes. Several studies have confirmed an association between unstable patterns and successful psychotherapeutic outcome. Somewhat similarly, the assimilation model of psychotherapeutic change argues that clinical change occurs through the integration of problematic experiences that initially threaten the stability of the self. This study examined how instability in assimilation levels was related to assimilation progress and change in symptom intensity, within and across sessions, in a good-outcome case of Emotion Focused Therapy. We used the assimilation of problematic experiences scales (APES) to measure assimilation and the outcome-questionnaire (OQ-10) to measure clinical symptom intensity. To assess assimilation instability, we used a fluctuation measure that calculated the amplitude and the frequency of changes in assimilation levels. To analyze the structural relationships between variables we used a dynamic factor model. The results showed that APES level and APES fluctuation tended to increase across treatment, while OQ-10 scores tended to decrease. However, contrary to expectations, the dynamic factor model showed no significant associations between APES fluctuation and OQ-10 scores either within sessions or between adjacent sessions.

## Introduction

Dynamic systems theory suggests that psychotherapeutic change requires periods of instability that permit the problematic state of maladaptive and rigid stability of the self to be transformed (Fisher et al., 2011). Due to therapeutic interventions, the self becomes permeable to new inputs that shift the system to a chaotic and instable state. The system then gradually changes to a more adaptive configuration as it returns to a state of homeostasis or stability (Fisher et al., 2011).

A similarly important role for instability can be derived within the assimilation model of psychotherapeutic change (Stiles, 2011). The assimilation model suggests that a person’s usual self is composed of traces of previous experiences, interconnected (*assimilated*) into aggregates, which can be reactivated. The traces, like the original experiences, incorporate intentions and intended actions as well as perceptions, cognitions, and feelings, so the aggregates can act and speak when they are reactivated, and they are characterized metaphorically as *voices*. Thus, we say a person is composed of the voices of the people, activities, skill, interests, and so on, that have comprised their life experience. Although most experiences are assimilated unproblematically, some problematic (e.g., traumatic, unacceptable, threatening, incongruent) experiences remain disconnected and liable to cause distress and dysfunctional behavior when they are reactivated. Or to say it another way, the disconnected voices emerge and cause problems when they are addressed by circumstances. Their being disconnected also prevents access to them, depriving the self of potential experiential resources. Psychotherapeutic change occurs through the assimilation of these disconnected, problematic internal voices.

The emergence of the problematic voices in therapy initially jeopardizes the stability of the self’s (maladaptive) state of functioning. The instability, manifested in the often difficult dialog within sessions, begins a progressive transformation of both parts through the creation of mutual understandings. In successful therapy, this process of assimilation promotes a more adaptive and functional self-structure and a return to a state of stability. In this way, instability plays an important role in the assimilation model’s account of change that is congruent with that of dynamic systems theory.

Instability during the assimilation process has been studied qualitatively through the analysis of setbacks (Caro Gabalda, 2006, 2008; Caro Gabalda and Stiles, 2013), and dynamic systems approaches have been used to study fluctuations in symptom intensity (Schiepek et al., 1997, 2013, 2014; Hayes et al., 2007a; Gumz et al., 2010; Heinzel et al., 2014). The present study examined the associations among assimilation, fluctuation in the assimilation process, and symptom intensity across sessions in a good outcome case of Emotion Focused Therapy using structural equation modeling (Fisher et al., 2011). To quantify the amount of instability in the assimilation process we measured the amplitude and frequency of setbacks using a measure of fluctuation designed to assess non-stationary phenomena in short time-series (Schiepek and Strunk, 2010).

This was a theory-building case study; that is, we aimed to analyze how the observations of this specific case were congruent with the assimilation model or, if not, suggested modifications or elaborations of the theory (Stiles, 2009). I theory-building case study research, as in most research in paradigmatic sciences, results such as detailed case observations bear on confidence in the theory. Substantive generalizations proceed from the theory, not directly from the results, so they do not depend on representative sampling (Stiles, 2009; Stiles et al., 2015).

### Dynamic Systems and Therapeutic Change

Studies within the Dynamic Systems approach have suggested that therapeutic change is associated with fluctuations or discontinuities in clinical symptoms (Kowalik et al., 1997; Schiepek et al., 1997, 2009, 2013, 2014; Hayes et al., 2007b; Ebner-Priemer et al., 2009; Schiepek, 2009; Gumz et al., 2010; Schiepek and Strunk, 2010). This is contrary to the usual expectation that a higher dose of treatment will result in progressively better outcome, or in other words, that there is a linear relation between input (dosage of treatment) and output (clinical outcome) (Schiepek et al., 2014). For example, in a study with depressive patients, Hayes et al. (2007b) found that, in some clients, the evolution of clinical symptoms assessed by standard measures was not regular but discontinuous and that this pattern of discontinuity predicted lower post-treatment scores of depression. Two discontinuous patterns were identified: early responders (a sudden gain in the first few sessions) and V-shaped patterns, or depressive spikes that occurred in an exposure-activation phase of treatment (Hayes et al., 2007b). Comparable results were found in a group of clients diagnosed with Obsessive Compulsive Disorder (OCD), where critical instabilities preceded important and significant transitions (Heinzel et al., 2014; Schiepek et al., 2014). Periods of instability within the client-therapist interaction were also associated with better outcome (Schiepek et al., 1997; Gumz et al., 2010).

The results of these studies suggest a view of the self as a dynamic system (Schiepek et al., 2014) in which instability is associated with flexibility, whereas stability is associated with order and rigidity (Fisher et al., 2011). When the organization of the system is too rigid, patterns of thoughts, feelings and behaviors are restricted, so that the individual may struggle to deal with new situations and may be vulnerable to clinical problems (Fisher et al., 2011). To promote more flexible and adaptive patterns of functioning, therapy has to introduce a bit of variability and chaos into the system (Fisher et al., 2011). According to Dynamics Systems approach, there may be transitions between one stable pattern of functioning and another, across the course of treatment (Gelo and Salvatore, 2016). First order change (also called within-order, stability-maintaining, or conservative change) refers to a pattern of relatively smooth, continuous changes occurring across therapy while maintaining the same dominant pattern of functioning (Gelo and Salvatore, 2016). Second order change (also called order-to-order or transformative change) refers to a significant and structural change from one previous pattern of functioning to a qualitatively different one. Usually, second order changes are characterized by periods of critical instability and fluctuations in specific therapeutic parameters (such as symptoms, alliance, etc.) (Gelo and Salvatore, 2016). These significant disruptive periods seem to be unique opportunities for change, since they allow clients to explore *“new, possibly more functional patterns of functioning*” (Gelo and Salvatore, 2016, p. 386). Significant changes in therapy seem to be preceded by periods of critical and abrupt instability and fluctuation. It seems that both stepping forward and stepping back are essential for successful psychotherapeutic change (Pascual-Leone, 2009).

### The Assimilation of Problematic Experiences

The assimilation model suggests that the voices that compose the self are assimilated to each other by semiotic meaning bridges, forming a community of voices (Honos-Webb and Stiles, 1998; Osatuke et al., 2005b). Voices emerge to speak for the community, usually voices representing past experiences that resemble the present situation in some way (Caro Gabalda and Stiles, 2009; Caro Gabalda, 2014). Flexibility in the organization of the community is needed to adapt to changing life events, and normally the voices to emerge and speak or act are appropriate to the requirements of the situation (Humphreys et al., 2005; Caro Gabalda, 2011). Voices representing new experiences are normally assimilated smoothly. However, the emergence of voices of experiences that threaten the community’s stability (e.g., traumatic incidents, destructive relationships, threatening or painful situations) are too dystphoric and are, in effect, rejected or ignored by the community. Their assimilation would require a significant change in the structure of the community. Nevertheless, such problematic voices try to speak when they are addressed by circumstances (Osatuke and Stiles, 2006). This encounter with the community produces strong negative feelings (Stiles et al., 2004). Theoretically, such disruptions help explain the affective aspects of psychological disorders, such as depression and anxiety.

In psychotherapy, clients can assimilate such problematic voices into the community (the self) through a dialog that creates semiotic meaning bridges, or mutual understandings, between them (Honos-Webb et al., 1999b; Stiles and Brinegar, 2007). The assimilation shifts the relation between voices, from conflict to understanding and joint action. It requires changes in both voices and a restructuring of the community into a more flexible and functional structure (Honos-Webb and Stiles, 2002). For example, in the case of Laura, a CBT client (drawn from the case study by Basto et al., 2017), her community was characterized as perfectionist, dominated by highly demanding voices that required perfection in every situation. Her problematic voice represented her experiences of failure in a variety of different intra- and interpersonal contexts. The conflict between the problematic voice’s need to be heard when it was addressed and the community’s need to hide this voice generated suffering and depression. The therapeutic dialog between the conflicting parts seemed to facilitate the assimilation of the problematic voice into the community and more generally the transformation of the community into a more flexible and less rigid structure. By the end of therapy, Laura could accept failure as a normal feature of her daily life, making her more resilient.

A series of intensive case studies on a variety different therapeutic models (e.g., Leiman and Stiles, 2001; Honos-Webb et al., 2003; Osatuke et al., 2005a, 2007; Brinegar et al., 2008; Mosher et al., 2008) has suggested that the assimilation of problematic experiences into the self progresses through a regular sequence, summarized in the eight levels assessed by the Assimilation of Problematic Experiences Scale (APES; see **Table 1**; Stiles et al., 1991; Stiles, 1999; Caro Gabalda and Stiles, 2009). A problematic experience’s assimilation into the self can evolve from level 0, where the problematic experience is completely out of awareness, to level 7 where the problematic experience is completely integrated into the self. Achieving higher APES levels during therapy is associated with good outcome (Detert et al., 2006; Basto et al., 2018).

**Table 1:** Assimilation of problematic experiences scale.

APES Level	Cognitive content	Emotional content
(0) Warded off/Dissociated	Content is unformed; client is unaware of the problem.	Distress may be minimal, reflecting successful avoidance.
(1) Unwanted thoughts/Active avoidance	Content includes distressing thoughts. Client prefers not to think about it.	Strong negative feelings.
(2) Vague awareness/Emergence	Client acknowledges his problematic experience and describes the distressing thoughts, but cannot formulate the problem clearly.	Feelings include acute psychological pain or panic.
(3) Problem statement/Clarification	Includes a clear statement of a problem, that is, something that could be worked on.	Feelings are mainly negative but manageable, not panicky.
(4) Understanding/Insight	The problematic experience is placed into a schema, formulated, understood, with clear connective links (meaning bridge).	There may mixed feelings with some unpleasant recognitions, but also with curiosity or even pleasant surprise.
(5) Application/Working through	The understanding is used to work on a problem, so there are specific problem-solving efforts.	Affective tone is positive and optimistic.
(6) Resourcefulness/Problem solution	Client achieves a solution for a specific problem. As the problem recedes, feelings become more neutral.	Feelings are positive, satisfied, proud of accomplishment.
(7) Integration/Mastery	Client successfully uses solutions in new situations, automatically.	Feelings are neutral because problem is no longer a problem.

Progress through different assimilation levels is not smooth but is characterized by frequent setbacks, defined as a return from a higher to a lower level of assimilation (Caro Gabalda and Stiles, 2013). Such setbacks have been observed in varied therapeutic approaches (Knobloch et al., 2001; Leiman and Stiles, 2001; Osatuke et al., 2005a; Caro Gabalda, 2006, 2008; Detert et al., 2006; Goodridge and Hardy, 2009; Caro Gabalda and Stiles, 2013). Setbacks are usually associated with transitions from one strand of a problem that is better assimilated to another strand that is not so well assimilated. Good outcome cases exhibit at least as many setbacks as poor outcome cases (Caro Gabalda, 2006; Mendes et al., 2016).

Setbacks represent a kind of destabilization or fluctuation. In light of the dynamic systems suggestion that destabilization can be therapeutically valuable, this suggested to us that it may be useful to find quantitative ways to assess this fluctuating pattern of assimilation progress.

### Purpose and Aims of This Study

We explored how fluctuation in APES levels was related to assimilation progress and change in symptom intensity within and across sessions in the case of a single good-outcome client. We used dynamic factor analysis, a technique designed for analyzing time-series data, especially in the analysis of interactions between response variables (Zuur et al., 2003). As Fisher and collaborators suggested “the degree of patterning in individual dynamic systems during psychotherapy can be modeled effectively via dynamic factor analysis” (Fisher et al., 2011, p. 554). To assess APES fluctuation, we used a measure, described later, that calculated the amplitude and the frequency of changes in assimilation levels (Schiepek and Strunk, 2010).

## Materials and Methods

### Client, Therapist, and Treatment

Alice (a pseudonym) was a single-26 year old employed woman who was treated for depression at the Maia University Institute (ISMAI), Portugal. She participated in a randomized clinical trial called the ISMAI Depression Study (Salgado, 2014), which compared the efficacy of Cognitive behavioral therapy (CBT) and Emotion Focused Therapy (EFT) for patients diagnosed with major depressive disorder.

Psychological treatment, as well as the collection and processing of data for research purposes followed principles and standards included in the ethics code (American Psychological Association’s – APA - Ethical Principles of Psychologists and Code of Conduct, as well as the Code of Ethics of Portuguese Psychologists). The client of this study, like all other participants, signed an informed consent required in the Standard 3.10 of the ethics code. Previously, the client was informed about the purposes of the research, expected duration and procedures. In addition, it was clarified that participation was voluntary, preserving their right to refuse participation or to give up participating at any time. In this informed consent, Alice also authorized the use of the collected data for process and outcome studies.

Alice received 16 weekly sessions of EFT and was considered a good-outcome client, as described later. She lived with her parents, who were catholic and conservative. Her presenting problems concerned her relationships with her boyfriend and with her parents (mainly her father) and problems at work. Mendes and collaborators (Mendes et al., 2016) previously classified categories of setbacks in this case.

Alice’s therapist was a Portuguese woman in her early thirties with 8 years of experience as a therapist, including 4 years of experience delivering EFT.

Emotion Focused Therapy is an empirically validated humanistic therapy (Greenberg, 2002; Elliott et al., 2004; Greenberg and Watson, 2006) that views emotions as an essential element in human functioning. According to this therapeutic model, emotions signal important needs underlying people’s experiences and promote adaptive action tendencies, helping them adapt and survive. Psychological problems are viewed as consequence of maladaptive emotional processing. The main therapeutic goal is to change this maladaptive emotional processing, allowing adaptive emotions to emerge and promote more adaptive functioning (Pos and Greenberg, 2007).

### Measures

#### Beck Depression Inventory-II (BDI-II)

The Portuguese version of the BDI-II (translated into Portuguese from Beck et al., 1996; Coelho et al., 2002) is a 21-item self-report questionnaire that assesses depressive symptoms. Higher total scores indicate severe depressive symptoms. For the Portuguese population, significant clinical depressive symptoms are signaled by a total score higher than 13. The results of the Portuguese validation were considered good (Coelho et al., 2002). Internal consistency reliability measured by Cronbach’s Alpha was 0.89 (Coelho et al., 2002).

#### Outcome Questionnaire-10 (OQ-10)

The OQ-10 (Lambert et al., 1998) is a self-report questionnaire composed by 10 items that measures health functionality. Each item is scored on a scale ranging from 0 to 4 and the total score goes from 0 to 40. Obtaining a higher score in this questionnaire indicates the presence of poorer mental health functionality. The OQ-10 Cronbach’s Alpha was of 0.88 (Seelert, unpublished) and the test–retest reliability of 0.62 (Lambert et al., 1998). In the sample from ISMAI Depression Study (*n* = 64; Salgado, 2014), the internal consistency was of 0.88 (Cronbach’s Alpha) and the test–retest reliability was of 0.74 over a 1-week interval.

#### Assimilation of Problematic Experiences Scale

As summarized in **Table 1**, the APES (Stiles et al., 1991; Caro Gabalda and Stiles, 2009) describes the evolution of the relation of a problematic experience (or voice) to the self (dominant community of voices) using a sequence of eight stages, numbered 0 to 7, ranging from warded off (i.e., muted or dissociated) to mastery (i.e., fully integrated and no longer a problem, serving as a resource in new situations). Theoretically, the APES is considered as a continuum, and intermediate ratings (e.g., 2.3, 4.6) are allowed. However, for this study, we used only whole numbers on the APES (e.g., 2, 3).

### Procedure

The BDI-II was administered at initial and post-treatment assessments and at sessions 1, 4, 8, 12. The OQ-10 was administered immediately before each session. Alice’s 16 sessions were videorecorded and later transcribed verbatim following the transcription conventions described by Mergenthaler and Stinson (1992).

#### Client Selection

Alice met criteria for the ISMAI Depression Study, which included: being diagnosed with Major Depression Disorder; Global Assessment of Functioning > 50. The exclusion criteria were: currently on medication or another form of treatment; or currently or previously diagnosed with one of the following DSM–IV Axis I disorders: panic, substance abuse, psychotic, bipolar, or eating disorder; or one of the following DSM–IV Axis II disorders: borderline, antisocial, narcissistic, or schizotypal; or at high risk of suicide. Screening for inclusion and exclusion criteria used the Structural Clinical Interview for the DSM-IV-TR (First et al., 1997, 2002). After being admitted to the study, each client was randomly attributed to a therapeutic condition (CBT or EFT) and, afterward, randomly assign to a therapist.

Alice’s scores on the BDI-II the ISMAI trial’s criterion measure, declined from 29 at initial assessment to 1 at her last session and 5 at 1-year follow-up. She was considered a good-outcome case because she met criteria for clinically significant and reliable improvement, as described by Jacobson and Truax (1991): (a) Her scores improved from above to below the cut-off of 13 on the Portuguese BDI-II, indicating clinically significant improvement, and (b) the amount of change was greater than the reliable change index of 7.75, that is, a difference greater than likely to have occurred by chance (at *p* < 0.05), indicating reliable improvement (Coelho et al., 2002). For this case study, she was selected from among the ISMAI trial clients who met the Jacobson and Truax (1991) criteria on the basis that complete transcripts were available.

#### Assimilation Analysis

Our assimilation analysis followed procedures used in previous studies (e.g., Stiles et al., 1991, 1992; Stiles and Angus, 2001; Honos-Webb et al., 2003).

The two APES raters were a PhD clinical psychologist, and a Ph.D. student in clinical Psychology, both with previous experience in research on the assimilation model. Training took approximately 4 months and included weekly meetings in which journal articles about the assimilation model were read and discussed and, sample sessions were coded according to the APES until all raters were considered reliable, achieving an intraclass correlation coefficient reliability of ICC [2,1] ≥ 0.60 (Cicchetti, 1994).

Next, both members of the team read transcripts of the entire case and identified the main recurring issues. By consensual agreement, they chose and characterized two main themes based on clinical relevance and time spent in therapy. The first theme was “fear of being rejected and abandoned,” which concerned Alice’s difficulty in imposing her needs to others, motived by an intense fear of not being accepted for what she was. The other theme selected was “hurt toward her father,” which concerned unfinished business with her father concerning an episode in which, at the age of 15, she discovered an affair her father was having. She had never confronted him about this, but she resented his infidelity. Alice’s dominant voice was labeled as “fear of being rejected,” as she presented a similar interpersonal pattern across contexts: work, relationships with family and boyfriend. The problematic voice for both themes was labeled as “I have the right to express myself and be accepted,” characterizing similar experiences of wanting to assert her needs and rights. These two voices seemed closely similar for both of these themes.

After selecting the themes, raters excerpted all passages where the themes appeared (*N* = 554) and rated them, independently according to the APES. The passage was the rating unit and was defined as a stretch of discourse delineated by a change in the topic of the conversation or by markers of changes in APES level (see Honos-Webb et al., 1999a, 2003). Reliability of these independent APES ratings was assessed using the Intraclass Correlation Coefficient designated ICC (2,2) by Shrout and Fleiss (1979), which is the reliability of the average of two raters. The ICC (2,2) was of 0.966, which is considered good (Cicchetti, 1994). Subsequently, raters discussed and reached consensus on APES ratings of passages where they disagreed (see Hill et al., 2005). The consensus ratings were used in our analyses.

### Analysis

#### Calculation of Mean APES Levels

**FIGURE 1:**
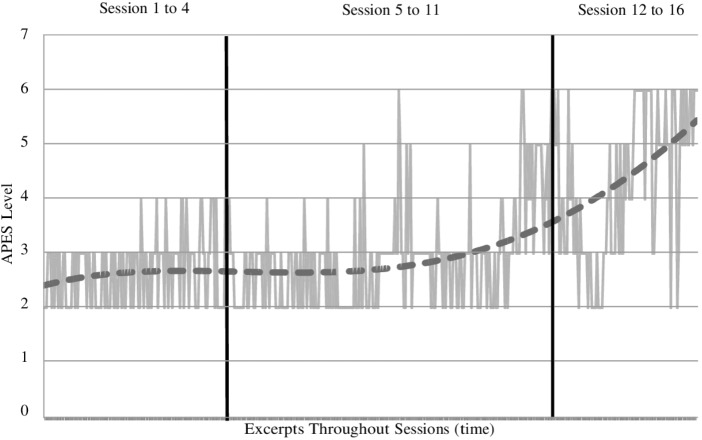
Assimilation progress of the “fear of being rejected and abandoned” theme across sessions.

**FIGURE 2:**
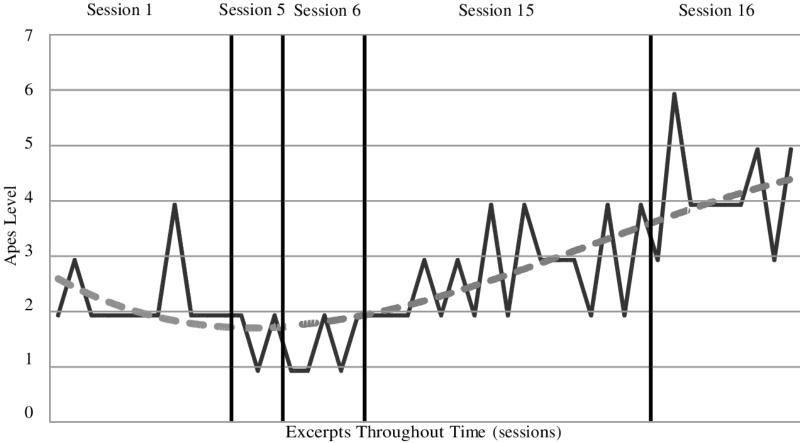
Assimilation progress of the “hurt towards her father” theme across sessions.

**Figures 1**, **2** show the assimilation progress in each theme, across passages. Passages dealing with particular themes were not evenly spaced across sessions, and the divisions shown in the figures are meant to indicate the approximate parts of the treatment in which each theme was addressed. The “fear of being rejected and abandoned” theme was much more frequent than the “hurt toward her father” theme. However, because both themes involved the same problematic and dominant voices, suggesting they were different expressions of a common core problem, we decided to combine these themes for our analysis. We also noted that the evolution of the two themes, considered separately, appeared very similar in terms of both APES levels and instability. Thus, to obtain mean APES levels for each session, we averaged APES ratings across passages of both themes within each session.

#### Index of Fluctuation in Assimilation Progress

Assimilation of problematic experiences scales fluctuation in each session was indexed using a measure fully described by Schiepek and Strunk (2010), which assessed the amplitude and the frequency of changes in APES levels across one session. In effect, the APES fluctuations index is a way to index the incidence of APES setbacks. Fluctuation values can vary from 0 (low fluctuation) to 1 (high fluctuation). That is, the formula yields a normalized fluctuation intensity, 0 ≤*F* ≤ 1:

$$F=\frac{\sum_{i=1}^{I}\frac{y_{i}}{\left(n_{k+1}-n_{k}\right)}}{s(m-1)}$$

where

*y_i_* = |*x_nk_* + 1 – *x_nk_*_j_

*x_n_ = n*th session score

*k =* points of return (changes in slope in the data sequence)

*i =* periods between points of return

*I =* total number of such periods within the window

*m =* number of measurement points within a moving window

*m* – 1 = number of intervals between all measurement points of a window

*s* = *x*_max_ – *x*_min_ with *x*_min_ smallest value of the scale, *x*_max_ largest value of the scale.

#### Dynamic Factor Analysis

In the present study we employed a dynamic factor model (Molenaar, 1985) that is a vector-autoregressive (VAR) method to measure contemporaneous correlations and time-lagged regressions in multivariate time series, using a structural equation model framework. The first step for this analysis was to smooth the time-series data and remove its trend. A moving average was calculated for the APES level, APES fluctuation, and symptom intensity measured by the OQ-10. The moving average was calculated by taking the arithmetic mean of a moving-window of four sessions. Total observations within each variable was equal to 16, the total number of sessions of Alice’s treatment. For the dynamic factor analysis, we treated each variable as a separated dependent variable.

A VAR model with a lag of 1 session was tested by creating a block-Toeplitz matrix. The VAR model allowed to analyze intra- and intersession relationships between each variable. All VAR analyses were carried out in LISREL (Version 8.80; Jöreskog and Sörbom, 1996). Since we had a single indicator for each latent variable, the lambda (λ) was set to identify and the beta (𝜃) matrix was fixed at zero. The beta matrix was then analyzed to search for meaningful modification indices (MIs). To improve our model, whenever there was a significant MI, we rerun the analysis with the significant path. The final model was accepted when no more significant MIs were observed within the matrix.

## Results

**Table 2** shows the values of mean APES level, APES fluctuation, and symptom intensity (OQ-10) for each of Alice’s 16 sessions. Alice’s OQ-10 scores were relatively high in the first three sessions, varied a good deal across the next ten sessions, and were relatively low in the final three sessions, consistent with her having achieved reliable and clinically significant improvement on the BDI-II. Her APES levels remained moderate through most of the therapy but were higher in the last four sessions. Her APES fluctuation, similarly, was relatively low until session 12 and then increased for the last five sessions.

**Table 2:** Comparison between the results of the variables clinical symptoms, assimilation and instability in Alice’s sessions.

Session	Symptom intensity (OQ-10 score)	Assimilation (mean APES rating)	Assimilation instability (APES Fluctuation)
S1	21	2.40	0.08
S2	19	2.54	0.08
S3	20	2.88	0.10
S4	12	2.77	0.08
S5	10	2.60	0.05
S6	15	2.25	0.08
S7	17	2.65	0.09
S8	14	2.28	0.07
S9	18	2.56	0.10
S10	19	3.14	0.08
S11	18	2.78	0.08
S12	16	4.71	0.13
S13	8	3.15	0.09
S14	6	4.61	0.10
S15	7	3.56	0.13
S16	7	4.97	0.14

The dynamic factor model measures of goodness of fit were satisfactory, including the chi-square value (χ^2^ = 6.84, *df* = 10), the root-mean-square-error of approximation (RMSEA = 0), the standardized root mean square residual (SRMR = 0.16) and the comparative fit index (CFI = 1).

**FIGURE 3:**
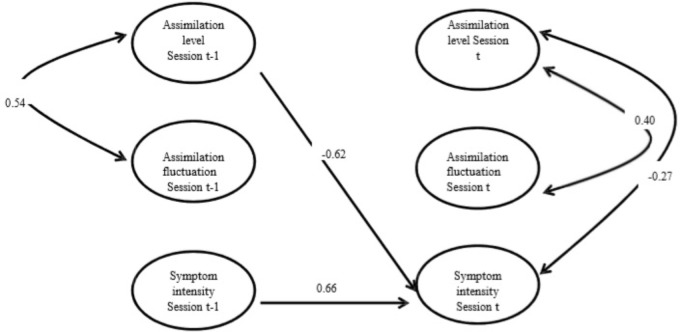
Dynamic Factor Model of Alice’s session-level APES levels, APES fluctuation, and Symptom Intensity.

**Figure 3** shows the dynamic factor model of Alice’s case. Within session (t-1) there was a positive association (0.54) between APES level and APES fluctuation. When the problem was at higher APES levels, APES fluctuation was also higher. Within session t, there was a similar positive association (0.40) between those variables, but also a negative association (-0.27) between OQ-10 scores and APES level. That is, when OQ-10 scores were relatively low, APES level was relatively high. Of particular interest, APES level in session t-1 negatively predicted OQ-10 scores at the following session (-0.62), that is, when a problem was at relatively higher assimilation levels in one session, OQ-10 scores decreased in the following session. Finally, not surprisingly, OQ-10 scores at session t-1 positively predicted OQ-10 scores at the following session (0.66).

## Discussion

In this good-outcome case, APES level and APES fluctuation tended to increase across treatment, while OQ-10 scores tended to decrease (**Table 2**). These changes were most apparent in the last four or five sessions of Alice’s 16-session treatment. The inverse associations of APES level with OQ-10 scores in the same session and the preceding session are consistent with assimilation theory. The direct association between APES level and APES fluctuations suggests that, at least in this case, instability increased as degree of assimilation rose. However, the dynamic factor model (**Figure 3**) showed no significant associations between APES fluctuation and OQ-10 scores either within sessions or between adjacent sessions.

The inverse association between APES level in one session and symptom intensity in the following session in Alice’s case was consistent with broader evidence that assimilation progress precedes therapeutic change as indexed by standard symptom intensity measures (Basto et al., 2018). Theoretically, the dialog between the problematic voice and the community promotes the assimilation of the problematic voice, and this assimilation underlies progress toward to a state of wellbeing. Becoming aware of and accepting previously avoided experiences overcomes the distress associated with encountering those experiences; it also gives access to experiential resources that were previously inaccessible to the self. Accepting diverse voices into the community promotes flexibility and a greater capability to adapt to diverse life situations.

At first sight, the failure of APES fluctuation to predict decreases in Alice’s OQ-10 scores in the subsequent session seems contrary to the dynamic systems perspective that instability may be necessary for therapeutic change. As shown in **Table 2**, fluctuation tended to be higher later in therapy, from session 12 to the last session, than in earlier sessions. We might speculate, based on Gelo and Salvatore’s (2016) suggestion about the role of instability in the promotion of therapeutic change, probably, that the lower instability up to session 11 could be reflect first order change, that is, relatively gradual, smooth, and continuous modifications around a dominant pattern of functioning. After session 12, the larger fluctuation values, might indicate the beginning of second order change, that is a more structural change in dominant pattern of functioning. Therefore, in Alice’s case, the instability phase seems to be associated with the systems re-organization to a more functional level. This speculation is congruent with the increasing APES values on assimilation and the decreasing OQ-10 scores after session 12. From a dynamic systems approach, second order changes would be associated with more substantial therapeutic change and, consequently, with a reduction in the intensity of clinical symptoms (Gelo and Salvatore, 2016). Thus, even though the reduction in clinical symptoms may not be linearly dependent on instability session by session, it may be promoted by periods of greater, more abrupt instability that will allow the systems reorganization into a more adaptive pattern. Our analysis would not have shown this association.

From this dynamic systems approach, then, a limitation of this study is that we tested if destabilization was associated with clinical symptom’s decrease only within the same session, or in the following session. If destabilization-improvement sequence occurs across multiple sessions, or in specific periods of the therapeutic process, then we might fail to detect it. For example, Hayes et al. (2007a) reported that destabilization pattern across many sessions would predicted symptom improvement only at post-treatment or follow-up. More studies are needed to test these alternatives.

From an assimilation model perspective, the potential therapeutic value of destabilizing may apply only when clients are rigidly denying or avoiding their problematic experiences, that is at APES levels 0 or 1, whereas even in her early sessions, Alice’s problems were at moderate APES levels, between APES 2, (vague awareness/emergence) and APES 3 (problem statement/clarification). Theoretically, the psychological rigidity–the denial and avoidance of problems – reflects the danger of powerful negative affect associated with encountering problems at low APES levels (Stiles et al., 2004; Basto et al., 2017). If the beneficial effect of destabilization predicted by dynamic systems approaches applies only to problems that begin at lower levels (below APES 2), it is understandable why the prediction did not work in Alice’s case.

More theoretically, perhaps APES fluctuation is not an appropriate measure of the sort of destabilization that is thought to promote change in dynamic systems. The previous work within the dynamic systems approach has focused on fluctuations in symptom intensity or distress. APES levels are related on symptom intensity (Basto et al., 2017), but they are expected to fluctuate with mood. As a different way to look at it, there is evidence that that large fluctuations in mood, or subjective distress are characteristic of the emotional upheavals of a problem’s passage through APES 2 (vague awareness/emergence), the emotional low point of the APES feelings curve (Mackay et al., 2002; Stiles et al., 2004). Perhaps passing through this emergence stage, with the accompanying emotional fluctuation, is the necessary step in therapeutic change.

Case studies such as this one can contribute to theory-building by their detailed correspondence or lack of correspondence with theoretical accounts and by showing where modifications in theory are required or where extensions are possible (Stiles, 2009). But of course, no case study is definitive. Further studies are needed to investigate the alternative possibilities we have raised.

## Author Contributions

IB designed and executed the study, assisted with the data coding and analyses, wrote the paper, and made a final approval of the version to be published; and agreed to be accountable for all aspects of the work in ensuring that questions related to the accuracy or integrity of any part of the work are appropriately investigated and resolved. WS made substantial contributions to the conception and design of the work, collaborated with the design, data analyses, and writing of the study; and editing of the final manuscript and made a final approval of the version to be published; and agreed to be accountable for all aspects of the work in ensuring that questions related to the accuracy or integrity of any part of the work are appropriately investigated and resolved. TB collaborated with the design, data analyses, and made a final approval of the version to be published; and agreed to be accountable for all aspects of the work in ensuring that questions related to the accuracy or integrity of any part of the work are appropriately investigated and resolved. PP and IM collaborated with the data coding and analyses, and writing of the study; and editing of the final manuscript and made a final approval of the version to be published; and agreed to be accountable for all aspects of the work in ensuring that questions related to the accuracy or integrity of any part of the work are appropriately investigated and resolved. DR collaborated in the writing of the study and editing of the final manuscript; and made a final approval of the version to be published; and editing of the final manuscript and agreed to be accountable for all aspects of the work in ensuring that questions related to the accuracy or integrity of any part of the work are appropriately investigated and resolved. JS collaborated with the design, data analyses, and writing of the study; and made a final approval of the version to be published; and editing of the final manuscript and agreed to be accountable for all aspects of the work in ensuring that questions related to the accuracy or integrity of any part of the work are appropriately investigated and resolved.

## Conflict of Interest Statement

The authors declare that the research was conducted in the absence of any commercial or financial relationships that could be construed as a potential conflict of interest. The reviewer PM and handling Editor declared their shared affiliation at the time of the review.
